# Abnormal level of CUL4B-mediated histone H2A ubiquitination causes disruptive HOX gene expression

**DOI:** 10.1186/s13072-019-0268-7

**Published:** 2019-04-16

**Authors:** Ye Lin, Juan Yu, Jianxin Wu, Shan Wang, Ting Zhang

**Affiliations:** 1 Beijing Municipal Key Laboratory of Child Development and Nutriomics, Capital Institute of Pediatrics, Beijing, 100020 China; 2 Graduate Schools of Peking Union Medical College, Beijing, 100730 China; 3 Department of Biochemistry and Molecular Biology, Shanxi Medical University, Taiyuan, 030001 Shanxi China; 4 Institute of Basic Medical Sciences, Chinese Academy of Medical Science, Beijing, 100730 China

**Keywords:** Neural tube defect, Histone ubiquitination, RA, CUL4B, RORγ, HOX genes

## Abstract

**Background:**

Neural tube defects (NTDs) are common birth defects involving the central nervous system. Recent studies on the etiology of human NTDs have raised the possibility that epigenetic regulation could be involved in determining susceptibility to them.

**Results:**

Here, we show that the H2AK119ub1 E3 ligase CUL4B is required for the activation of retinoic acid (RA)-inducible developmentally critical homeobox (HOX) genes in NT2/D1 embryonal carcinoma cells. RA treatment led to attenuation of H2AK119ub1 due to decrease in CUL4B, further affecting HOX gene regulation. Furthermore, we found that CUL4B interacted directly with RORγ and negatively regulated its transcriptional activity. Interestingly, knockdown of RORγ decreased the expression of HOX genes along with increased H2AK119ub1 occupancy levels, at HOX gene sites in N2/D1 cells. In addition, upregulation of HOX genes was observed along with lower levels of CUL4B-mediated H2AK119ub1 in both mouse and human anencephaly NTD cases. Notably, the expression of HOXA10 genes was negatively correlated with CUL4B levels in human anencephaly NTD cases.

**Conclusions:**

Our results indicate that abnormal HOX gene expression induced by aberrant CUL4B-mediated H2AK119ub1 levels may be a risk factor for NTDs, and highlight the need for further analysis of genome-wide epigenetic modifications in NTDs.

**Electronic supplementary material:**

The online version of this article (10.1186/s13072-019-0268-7) contains supplementary material, which is available to authorized users.

## Background

Neural tube defects (NTDs) are a group of severe congenital malformations caused by the failure of neural tubes to close completely. Human NTDs are a complex condition impacted by multiple factors, including both genetic and environmental. Recently, a study showed that the formation of the neural tube is under precise spatiotemporal control by cooperative actions between environmental factors and intrinsic signal transduction [1]. In humans, the development of NTDs is thought to involve the interplay of genes in the fetus and the effects of environmental factors. However, the molecular mechanisms in the etiology of NTDs through which environmental factors affect epigenetic regulation, thereby influencing the expression of susceptibility genes, are poorly understood.

Retinoic acid (RA), a derivative of vitamin A (retinol), is an extrinsic signal essential for neuronal differentiation in embryonic development [2, 3]. Paraxial mesoderm surrounding the neural tube expresses the retinaldehyde dehydrogenase-2 (Raldh2) enzyme, which converts retinaldehyde to RA, serving as a ligand for nuclear RA receptors that directly regulate gene expression at the transcriptional level [4]. It has been demonstrated that RA plays an essential role in anteroposterior patterning of neuroectoderm in the central nervous system (CNS) [5–8]. It is found that epigenetic change occurs in response to RA and is related to cell differentiation. The observation of histone enzymes involved in transcription of response genes, and many of these enzymes are modulated by RA treatment. Currently, some of these transcription complexes regulate histone modification to influence stem cell fate. Polycomb group complexes (PRC) play critical regulatory roles that change the expression of numerous genes during neuronal cell specification. Two main polycomb group complexes exist in mammals: PRC1 and PRC2. PRC1 catalyzes histone H2AK119 monoubiquitylation (H2AK119ub1). More recent studies have suggested that PRC1 plays an important role in H2AK119ub1 and Hox gene silencing. CUL4B is an important member of the Cullin–Ring ligase (CRL) complex, which has E3 ubiquitin ligase activity. It participates in many developmental processes, such as cell cycle progression, replication, and the DNA damage response and involves in the proliferation and organization of neuronal cells. Moreover, CUL4B might also be involved in the regulation of appropriate brain development. Recently, it was indicated that CUL4B possesses an intrinsic transcription repressive activity by promoting H2AK119ub1. CUL4B regulates transcription via H2AK119ub1 in coordination with H3K27me3 leading to derepression of target genes that are critically involved in cell growth and migration. Nuclear receptors constitute a superfamily of ligand-dependent transcription factors that includes receptors for retinoids and orphan receptors. In this study, screening of CUL4B targets by IP/mas identified the ROR γ as a specific substrate of CUL4B. The retinoid-related orphan receptors RORα, RORβ, and RORγ are a distinct subfamily of nuclear receptors. RORγ is an orphan nuclear receptor related to retinoic acid. RA can bind to RORγ as a ligand to produce biological effects. RA and synthetic retinoic acid ALRT1550 can bind to RORγ, thereby stimulating RORγ-mediated transcriptional activation [9]. Recent studies have demonstrated that RORs function as ligand-dependent transcription factors. Transcriptional regulation by RORs is mediated by the recruitment of corepressor and coactivator complexes modulating the response genes. It has been showed RORγ played major roles in neural stem cells development and neurological diseases. Although several studies have indicated that RA signaling is involved in neuron specification and neurogenesis, the molecular mechanisms of action associated with the target genes of RA-bound RORs during neural development have remained elusive.

Recently, more than 200 candidate genes have been identified as potentially being related to NTDs in humans. A prominent focus has been on the PCP, BMP, and Wnt pathways and on homeobox genes (Hox). Hox genes play an important role in brain and spinal cord development during establishment of the anteroposterior body axis in embryogenesis. In mammals, 39 Hox genes have been identified, which are divided into four clusters: A, B, C, and D. During embryonic development, the conserved Hox genes are clustered and expressed linearly, resulting in the formation of the body shape. Hox genes also play important roles in specifying the morphology of the vertebrae [10]. Hoxa1 plays an important role in regulating nerve ridge function, hindbrain formation, and early development [11]. Hoxa1 is also necessary for the RA-induced differentiation of embryonic stem cells (ESC) into neurons [12]. In addition, Hoxa7 participates in regulating cell differentiation [13] and the process of embryogenesis [14]. Moreover, Hoxa9 plays a role in regulating the differentiation of pluripotent progenitor cells [15], while Hoxa10 can regulate the expression of region-specific genes [16]. Hoxb1 regulates the proliferation of neural stem cells and progenitor cells [17]. The regulation of Hoxb7 over the course of development is time dependent and tissue specific [18]. In addition, it has recently been shown that some of the Hox genes play roles in global patterning in vertebral development. For example, several studies have indicated that RA induces the expression of Hox genes needed for rhombomeric segmentation of neuroectoderm during neural tube formation and spinal cord development [19]. It has been shown that RA signaling is necessary to erase polycomb repressive (PRC) activated Hox genes during embryonic stem cell differentiation [2]. However, the molecular mechanism by which CUL4B catalyzes H2AK119ub1 and contributes to RA-mediated HOX gene regulation in NTDs remains unclear.

To obtain a better understanding of the molecular mechanisms underlying CUL4B’s involvement in RA-mediated HOX gene regulation in NTDs, we here show that CUL4B is required for the activation of retinoic acid (RA)-inducible developmentally critical homeobox (HOX) genes in NT2/D1 cells. RA treatment led to attenuation of H2AK119ub1 due to a decrease in CUL4B, further affecting HOX gene regulation. Our study also identifies CUL4B as a novel repressor of RORγ transcriptional regulation. Knockdown of RORγ decreased the expression of HOX genes, along with increased H2AK119ub1 occupancy levels at HOX gene sites in N2/D1 cells under RA treatment. In addition, upregulation of HOX genes was observed, along with lower levels of H2AK119ub1, in both mouse and human anencephaly NTD cases. Together, our findings highlight how RA affects CUL4B and cofactor RORγ through histone modification, leading to the regulation of HOX gene transcription during the early stage of development. Our work also reveals the need for further genome-wide analysis of epigenetic modifications in NTDs.

## Results

### CUL4B represses transcription of HOX genes in RA-induced human NT2 cells

**Fig. 1:**
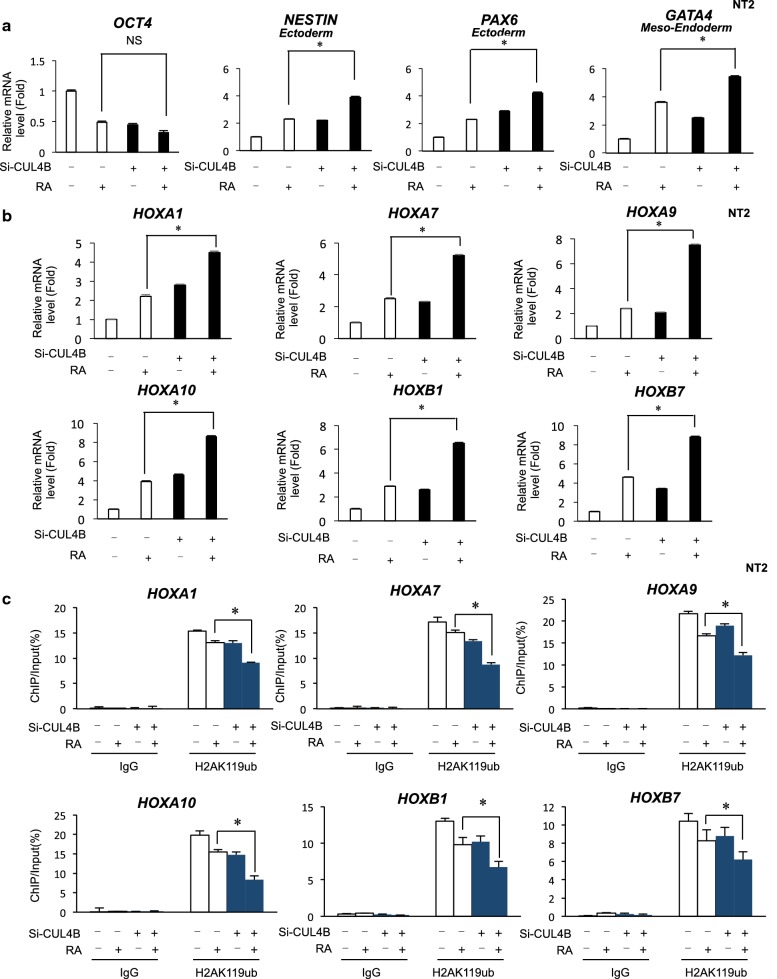
**a** Quantitative RT-PCR analysis showed that CUL4B loss had a positive effect on the RA-induced increases in differentiation genes mRNA levels in NT2 cells. Data were shown as mean ± SD (*n* = 3). *P < 0.05. **b** Knockdown of CUL4B affected mRNA level of Hox genes in RA-induced NT2 cells. NT2 cells were knockdown of CUL4B for 24 h. Then, after 24 h of RA treatment, cells were collected and analyzed. Data were shown as mean ± SD (*n* = 3). *P < 0.05. **c** Quantitative ChIP analysis was performed to analyze the effect of CUL4B loss on the RA-induced changes in H2AK119ub1 levels at the Hox genes. Enrichment of the Hox genes promoter was measured by qPCR. Data were shown as mean ± SD (*n* = 3). *P < 0.05

RA induces differentiation, proliferation arrest, and apoptosis in many cell types, including NT2 human embryonic carcinoma cells. CUL4B is an important component of the Cullin4B–Ring E3 ligase family. It plays a role in many physiological and pathological conditions, such as posttranslation modification, cell differentiation, cell cycle regulation, and DNA damage repair. We investigated whether CUL4B expression affected the RA-induced differentiation of NT2 cells toward parietal endoderm. NT2 cells were knocked down with CUL4B and differentiated under treatment with RA. CUL4B loss in NT2 cells did not affect RA-induced decrease in the expression of the pluripotency gene Oct4, but induced the RA-driven activation of the ectoderm marker gene Nestin, Pax6, and Notch1. CUL4B loss also increased the RA-driven activation of the mesoderm marker gene GATA4, GATA6, and Pax3, with increased basal expression in RA-untreated cells with CUL4B knockdown (Fig. 1a and Additional file 1: Fig. S1a). These results imply that loss of Cul4B and RA treatment leads to higher expression of these markers which suggest interactions between these two pathways in lineage determination. Together, CUL4B inhibits ectoderm and mesoderm lineage during RA-driven differentiation. Several studies have demonstrated that RA treatment induced the differentiation of teratocarcinoma and embryonic stem (ES) cells. During this differentiation process, there appears to be a collinear activation of Hox genes. We therefore tested whether CUL4B is associated with the levels of HOX gene expression in NT2/D1 cells. As shown in Fig. S1b, HOXA1, HOXA7, HOXA9, HOXA10, HOXB1, and HOXB7 genes were upregulated in CUL4B-depleted NT2 cells (Additional file 1: Fig. S1b). We next investigated whether CUL4B is involved in the RA-induced reduction of H2AK119ub1 levels at HOX genes in NT2/D1 cells. The inhibition of CUL4B expression by siRNA led to a strong increase in the levels of mRNAs encoded by HOX genes both before and after RA treatment (Fig. 1b). Quantitative analysis of the mRNA levels indicated that the HOX genes reported to be key regulators of NT2 differentiation are RA-dependent CUL4B targets. Previous results showed that the H2AK119ub levels are associated with the modulation of promoters of Hox genes [3]. ChIP analysis of selected promoters in the NT2 cells was performed after transfection with siRNAs targeting CUL4B using H2AK119ub antibodies. The levels of H2AK119ub1 were markedly decreased at HOXA1, HOXA7, HOXA9, HOXA10, HOXB1, and HOXB7 genes upon CUL4B knockdown cells (Additional file 1: Fig. S1c and S1d). We next evaluated how CUL4B knockdown affects the promoter activity of HOX genes for the RA-induced differentiation of NT2 cells. ChIP analysis showed a decrease in the level of H2AK119ub1 at the HOX gene promoter due to CUL4B siRNA both before and after RA treatment. Specifically, the expression of HOXA1, HOXA7, HOXA10, and HOXB1 was compromised upon CUL4B depletion in RA-treated cells (Fig. 1c). Thus, RA treatment led to decreased CUL4B expression and subsequently an attenuation of H2AK119ub1 binding to the promoters of HOX genes. This suggests that stimulation with RA leads to the dissociation of more CUL4B-containing complexes to HOX genes, which results in the reduction of H2AK119ub1. Taken together, these findings provide evidence indicating that CUL4B suppresses transcription of HOX genes in RA-induced human NT2/D1 cells.

### RA-induced Hox genes expression correlates with a decreased level of promoter H2AK119ub1 in mouse ESCs

**Fig. 2:**
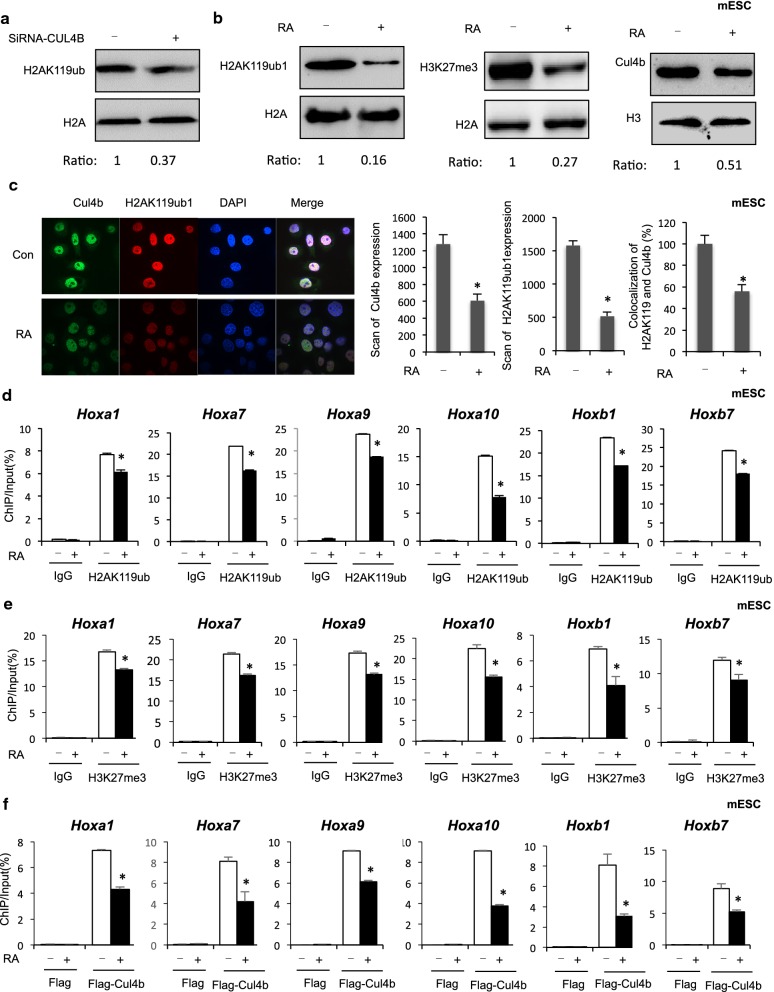
**a** Knockdown of CUL4B affected histone H2AK119ub1 modification level in NT2 cells. **b** Comparison of histone H2AK119ub1 modification level and Cul4b level between normal and RA-treated mouse ESCs by Western blotting. **c** The colocalization of Cul4b and H2AK119ub1 in the mouse ESCs cells with RA treatment. Direct immunofluorescence analysis was performed. Images were captured by confocal microscope, and the nuclei were stained with DAPI. **d** ChIP assays of H2AK119ub1 were performed using mouse ESC after RA treatment; mouse IgG was used as control. Enrichment of the Hox genes promoter was measured by qPCR. Data were shown as mean ± SD (*n* = 3). *P < 0.05. **e** ChIP assays of H3K27me3 were performed using mouse ESC after RA treatment; mouse IgG was used as control. Enrichment of the Hox genes promoter was measured by qPCR. Data were shown as mean ± SD (*n* = 3). *P < 0.05. **f** ChIP assays of Cul4b were performed using mouse ESC after RA treatment; mouse IgG was used as control. Enrichment of the Hox genes promoter was measured by qPCR. Data were shown as mean ± SD (*n* = 3). *P < 0.05

CUL4B functions as a histone modification enzyme responsible for H2AK119ub1, providing a molecular basis for the interplay of H2AK119ub1 and H3K27me3 in chromatin remodeling. Previous finding showed that CUL4B is found in a complex with PRC1 and PRC2; this indicates that RA-induced differentiation results in the dissociation of a complex exhibiting histone H2A ubiquitin ligase activity that mediates repression of the HOX promoters. Consistent with the previous results, knockdown of Cul4b decreased the level of H2AK119ub1 (Fig. 2a). Recently, it showed that the expression of HOX genes is tightly regulated during RA-induced differentiation of human NT2 cells, and that is associated with the rapid modulation of H2AK119ub1 levels at the promoters of HOX genes. We measured the expression of mRNA encoded by Hox genes expression in RA-treated mouse ESCs. The results showed that RA treatment led to a strong increase in the mRNA levels of Hoxa1, Hoxa7, Hoxa9, Hoxa10, Hoxb1, and Hoxb7 genes in mouse ESCs (Additional file 1: Fig. S2a). Genome-wide results from a previous study revealed RA-induced epigenetic changes in mouse embryo stem cells (ESCs) [2]. We found a decrease in the H2AK119ub1 and H3K27me3 level in RA-treated mouse ESCs (Fig. 2b, left and middle panel). This indicates that a suppressed level of H2AK119ub1 is likely to be associated with elevated Hox gene expression. Previously, it was shown that CUL4B possesses intrinsic transcriptional repression activity by promoting H2AK119ub1 [3]. We were thus interested in exploring the possibility that the decreased level of H2AK119ub in RA-treated mouse ESCs was due to the repression of Cul4b, a key factor for embryonic development. A significant decrease in the level of Cul4b expression was observed in RA-treated mouse ESCs (Fig. 2b, right panel). We also found that Cul4b was decreased in foci localized to the nucleus after exposure to RA and significantly downregulated of colocalized with H2AK119ub1 (Fig. 2c). As the first step to elucidate the regulation of H2AK119ub1 on Hox genes in RA treatment mouse ESCs, ChIP assays were performed to evaluate the binding of H2AK119ub1 to these six selected Hox genes (Hoxa1, Hoxa7, Hoxa9, Hoxa10, Hoxb1, and Hoxb7). As shown in Fig. 2d, e and Additional file 1: Fig. S2b and S2c, H2AK119ub1 and H3K27me3 binding to the sequences of Hox genes (Hoxa1, Hoxa7, Hoxa9, Hoxa10, Hoxb1, and Hoxb7) were reduced after RA treatment. The most significant attenuation was observed for binding to Hoxa10, for which a 50% decrease was observed. By contrast, no change in binding of H2AK119ub1 and H3K27me3 to the IgG locus sequences was observed (Fig. 2d, e). We then investigated the regulation of Cul4b on Hox genes in RA treatment. ChIP assays showed that Cul4b binding to the sequences of Hox genes (Hoxa1, Hoxa7, Hoxa9, Hoxa10, Hoxb1, and Hoxb7) was reduced after RA treatment (Fig. 2f). These results suggested that the association of elevated Hox genes expression with suppressed H2AK119ub in RA-induced mouse ESCs.

### CUL4B interacts with RORγ and alterations in HOX genes expression in human NT2/D1 cells

**Fig. 3:**
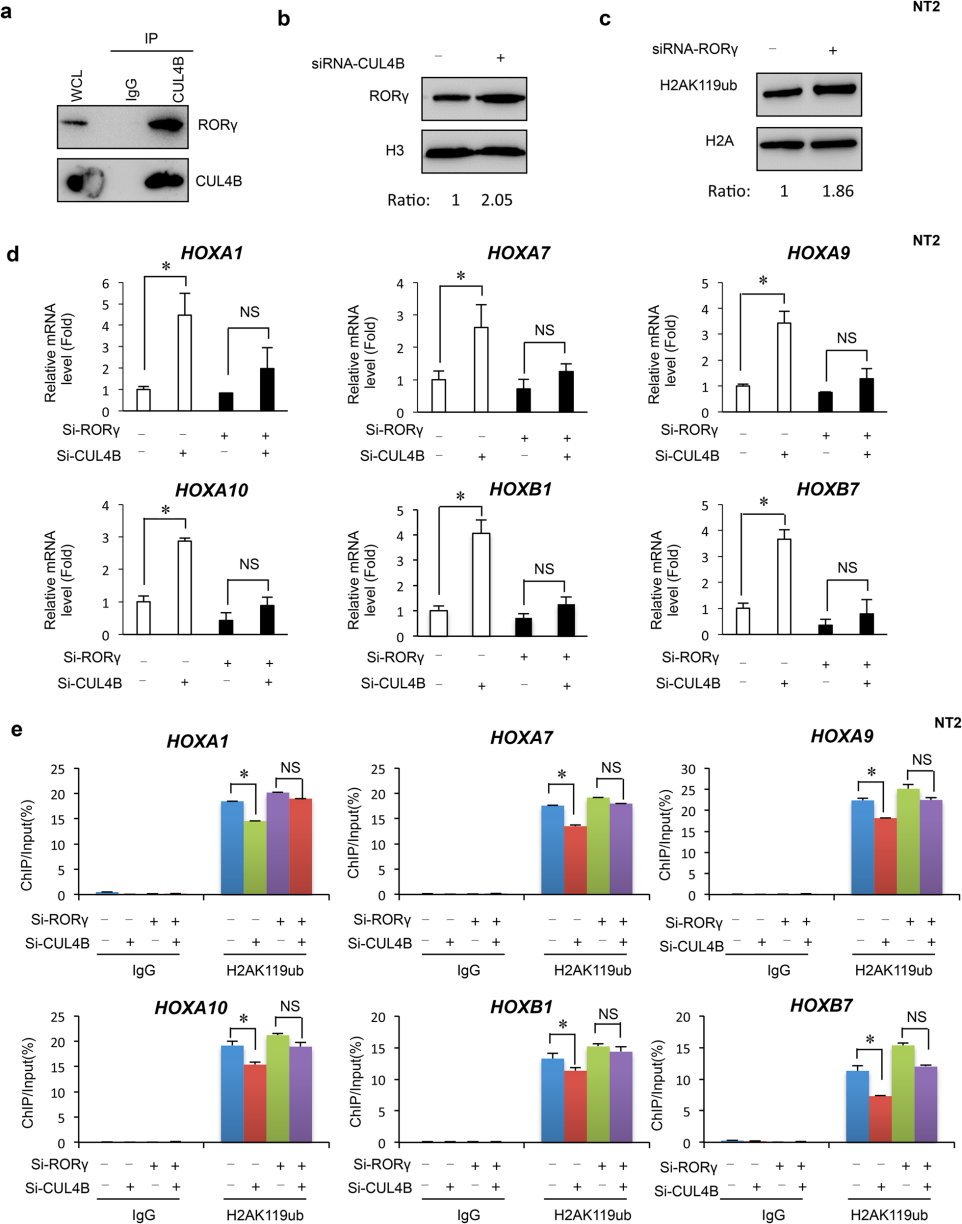
**a** The whole NT2 cell extract was immunoprecipitated with anti-CUL4B antibody and normal rabbit IgG antibody, and analyzed by western blot with anti-CUL4B and anti-RORγ antibodies. **b** Expression level of RORγ was measured by Western blotting after knockdown of CUL4B. **c** Expression level of H2AK1119ub1 was measured by Western blotting after knockdown of RORγ. **d** Quantitative RT-PCR analysis showed that CUL4B loss and RORγ had a negative effect on HOX genes in NT2 cells. Data were shown as mean ± SD (*n* = 3). *P < 0.05. **e** ChIP assays of H2AK119ub1 were performed using NT2 cells after knockdown of CUL4B and RORγ; IgG was used as control. Enrichment of the HOX genes promoter was measured by qPCR. Data were shown as mean ± SD (*n* = 3). *P < 0.05

In an effort to better understand the mechanistic role of CUL4B, we used mass spectrometry to identify the proteins associated with CUL4B in vivo. IP/Mas analysis using an antibody for CUL4B identified it as a potential RORγ-interacting protein and analysis of enrichment of GO and KEGG pathway (Additional file 1: Fig. S3a and Additional file 2: Table S1, Additional file 3: Table S2, Additional file 4: Table S3). Co-immunoprecipitation analysis with anti-CUL4B antibody also indicated that RORγ interacts with CUL4B (Fig. 3a). We further observed that the level of RORγ protein was increased when CUL4B was knocked down, whereas the stability of CUL4B protein was not significantly affected by RORγ (Fig. 3b and Additional file 1: Fig. S3b). Interestingly, we also found that the level of H2AK119ub1 was increased after RORγ knockdown (Fig. 3c). These experiments revealed that CUL4B’s interaction with RORγ might involve epigenetic regulation. We next examined whether RORγ is involved in the regulation of HOX genes. We have performed a series of experiments to investigate the possible important role by interaction of Cul4B with RORγ. We measured the mRNA levels of HOX genes using quantitative real-time RT-PCR with the knockdown of endogenous RORγ in NT2 cells. The knockdown of RORγ significantly reduced the levels of HOXA1, HOXA10, HOXB1, and HOXB7, but had only weak effects on HOXA7 and HOXA9 (Additional file 1: Fig. S3c). Interestingly, knockdown of CUL4B has no significant effects on HOX genes in deletion of RORγ cells. It suggested that RORγ was required for Cul4b to repress HOX efficiently (Fig. 3d). To further verify whether there are effects of RORγ on H2AK119ub1 binding to the HOX genes, we performed quantitative ChIP assays by real-time PCR using control IgG or H2AK119ub1 antibody. Knockdown of RORγ induced occupancy of H2AK119ub1 at HOX genes (Additional file 1: Fig. S3d). However, knockdown of CUL4B also has no significant effects on H2AK119ub1 binding to the sequences of HOX genes in deletion of RORγ cells (Fig. 3e). These findings suggest a model in which CUL4B binds to RORγ and involves in H2AK119ub1 binding to HOX genes. This implies that CUL4B interacts with RORγ and alterations in HOX genes expression in human NT2/D1 cells.

### RORγ is required for activation of HOX genes expression during RA-induced differentiation human NT2/D1 cells

**Fig. 4:**
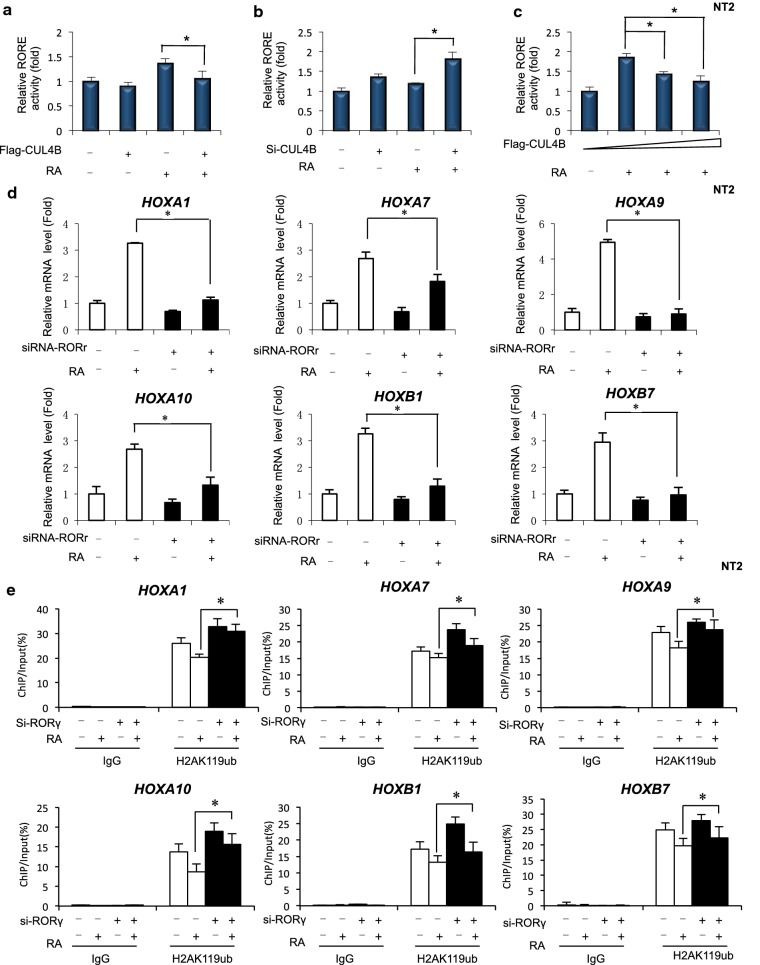
**a** NT2 cells were transfected overexpression of CUL4B. Thirty-six hours after transfection, RORE luciferase activity was measured after treatments with RA (1uM) for 12 h. Data are mean ± SD (*n* = 3). **b** NT2 cells were transfected with the siRNA-CUL4B. Thirty-six hours after transfection, RORE luciferase activity was measured after treatments with RA (1uM) for 12 h. Data are mean ± SD (*n* = 3). **c** NT2 cells were transfected with the increasing amounts of CUL4B. Thirty-six hours after transfection, RORE luciferase activity was measured after treatments with RA (1uM) for 12 h. Data are mean ± SD (*n* = 3). **d** Knockdown of RORγ affected mRNA level of Hox genes in RA-induced NT2 cells. NT2 cells were knockdown of ROR for 24 h. Then, after 24 h of RA treatment, cells were collected and analyzed. Data were shown as mean ± SD (*n* = 3). *P < 0.05. **e** Quantitative ChIP analysis was performed to analyze the effect of RORγ loss on the RA-induced changes in H2AK119ub1 levels at the Hox genes. Enrichment of the Hox genes promoter was measured by qPCR. Data were shown as mean ± SD (*n* = 3). *P < 0.05

To determine whether CUL4B modulates the transcriptional activity of RORγ by binding to RORγ, we expressed Flag-CUL4B with a luciferase reporter plasmid, RORE-responsive element of endogenous RORγ activity. Overexpression of CUL4B inhibited RORγ activity in NT2 cells upon RA treatment (Fig. 4a). Knockdown of endogenous CUL4B by siRNA significantly enhanced RORγ activity in NT2 cells in RA-treated cells (Fig. 4b). The coexpression of CUL4B inhibited RORγ activity in a dose-dependent manner (Fig. 4c). Next, we investigated whether RORγ involved in the RA-induced activation of HOX genes in NT2/D1 cells. The results showed that knockdown of RORγ led to a decrease in the levels of mRNAs encoded by HOXA1, HOXA7, HOXA9, HOXA10, HOXB1, and HOXB7 genes after RA treatment (Fig. 4d), as well as an increased level of H2AK119ub1 at HOXA1, HOXA7, HOXA9, HOXA10, HOXB1, and HOXB7 genes after RA treatment was observed (Fig. 4e). Recent studies have shown a relationship between the ROR regulatory network and the controls of metabolic homeostasis and development. This was supported by ChIP analysis showing that the level of H2AK119ub1 associated with these HOX gene sites was considerably higher in cells in which RORγ was downregulated. These observations suggest that the recruitment of RORγ to regulatory regions correlates with a less closed chromatin structure consistent with the inducing function of RORγ. Together, our findings indicate that RORγ may participate in HOX gene transactivation during RA-induced differentiation in human NT2/D1 cells.

### Cul4b level downregulated in RA-induced mouse NTD model

**Fig. 5:**
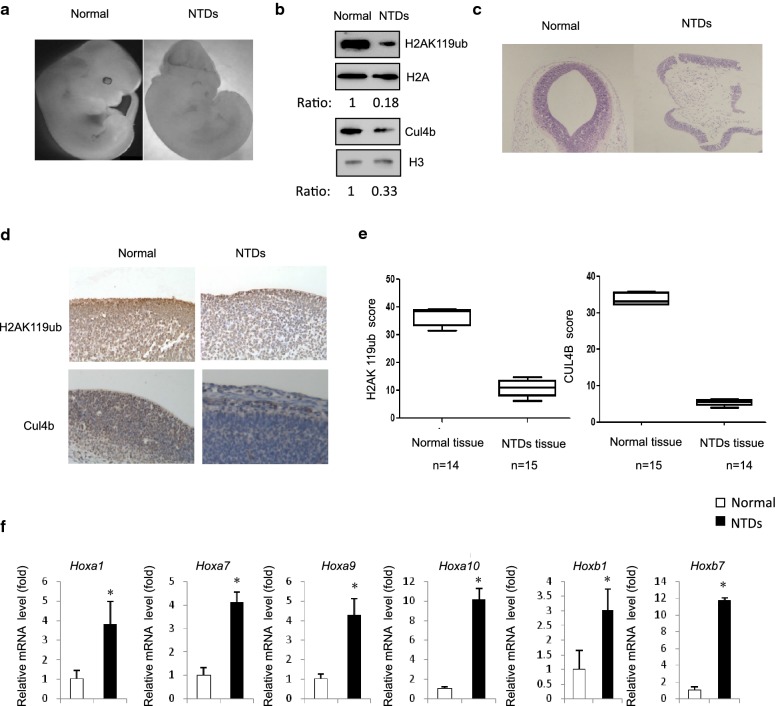
**a** RA-induced anencephaly in C57BL/6 mouse embryos at 10.5 day. **b** Cranial neural tissue of normal and RA-induced mouse NTDs was harvested at E13.5 and analyzed H2AK119ub1 and Cul4b by western blotting. Aliquots of total lysates were immunoblotted to indicate antibody. **c**,** d** Representative images from immunohistochemical staining of H2AK119ub1 and Cul4b in the cranial neural tissue. Scale bars, 100 mm. **e** H2AK119ub1 and CUL4B expression scores are shown as box plots, with the horizontal lines representing the median. Data are mean ± SD. (*n* = 5),*P < 0.05, by Student’s *t* test. **f** Hox genes mRNA in cranial neural tissue of RA-induced mouse NTDs was measured by RT-qPCR. Data are mean ± SD (*n* = 3)

**Fig. 6:**
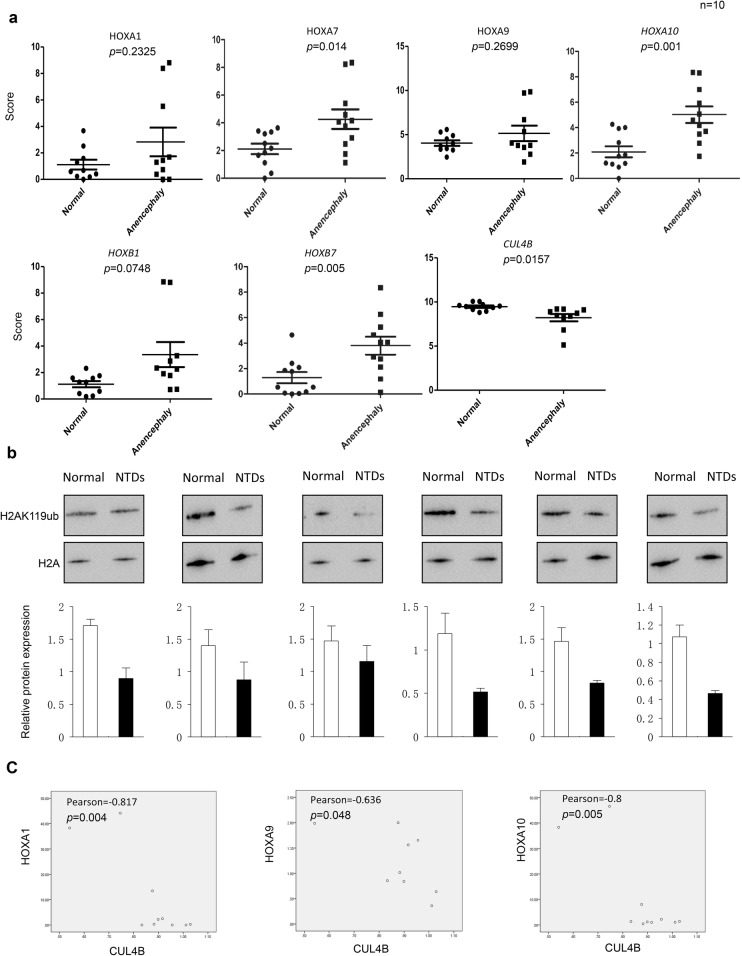
**a** The mRNA expression of CUL4B and Hox genes in the brain of NTD fetuses, determined by NanoString. Data are mean ± SD (*n* = 10),*P < 0.05, by Student’s *t* test. **b** Detection of H2AK119ub1 in brain tissues from human anencephaly and normal cases by western blotting. Total histone GAPDH was used as a loading control. Data are mean ± SD (*n* = 3),*P < 0.05, by Student’s *t* test. **c** Pearson’s correlation analysis between H2AK119ub1 expression and HOXA1, HOXA9, and HOXA10 expression

RA is thought to be involved in neurulation and subsequent neural tube patterning. Specifically, it is well known that RA functions in the regulation of Hox patterning genes. Previous studies showed that RA induces caudal neural plate explants fated to become posterior spinal cord to change Hox expression from a posterior to an anterior location [20–24]. RA is also a well-known teratogen, with its administration to embryos having been shown to induce neural tube defects (NTDs) [25–27]. Our previous data suggest that Hox gene expression pattern changes in RA-induced mouse NTDs model. To further define the role of cu14b correlated with Hox genes expression in neural development, we established a modified rapid RA-induced NTD mouse model via gavage of excess RA (25 mg/kg) at E7.5 [28]. As shown in Fig. 6a, control mice were exhibited complete organization, normal size, and an orderly arrangement of neuroepithelial cells. In contrast, in the RA-treated group, mouse embryos showed abnormal brain findings, such as defective spinal morphology (Fig. 5a). We next explored the possibility that the level of Cul4b in RA-induced cranial neural tissue from E13.5 embryos. The Cul4b level was remarkably decreased in RA-induced cranial neural tissue (Fig. 5b). Next, we were interested to explore whether there is decrease in the level of H2AK119ub1 in RA-induced mouse NTD embryos. The results showed that level of H2AK119ub1 was decreased in RA-induced cranial neural tissue from E13.5 embryos (Fig. 5b). We also investigated whether the expression of Cul4b and H2AK119ub1 was abnormally altered in cranial neural tissue. To this end, we examined Cul4b and H2AK119ub1 expression levels in 15 pairs of RA-induced cranial neural tissue samples and their matched normal tissues by immunohistochemical (IHC) analysis. The staining of total Cul4b and H2AK119ub1 decreased in mouse NTD samples compared with that in their normal tissues (Fig. 5c, d). The results showed that the downregulation of H2AK119ub was nearly 75% and that of Cul4b was 85% (Fig. 5e). The findings from the timed inhibition of RA activity demonstrated that RA aligns the neural and mesodermal tissue early during gastrulation, before the specification of hindbrain and spinal cord regions, and the expression of Hox patterning genes. We next evaluated the change in the level of expression of Hox genes at E13.5. We performed qPCR assays on cranial neural tissue of E13.5 mouse embryos; the results indicated that the mRNA levels of Hox genes were significantly increased in NTD embryos compared with those in controls, which was in accordance with the qPCR results for mESCs (Fig. 5f). Together, these results indicate that the downregulation of Cul4b expression is related to the occurrence of RA-induced mouse NTDs, probably through regulating alternative Hox signaling activity.

### HOXA10 gene transcription is altered with decreased CUL4B level in NTD fetuses

Patterning of the growing neural tube occurs in both the rostrocaudal axis and the dorsoventral axis under the influence of extracellular morphogens such as RA, Wnts, sonic hedgehog, and BMP signaling. Failure to complete closure of the neural tube at the rostral or caudal end leads to anencephaly and spina bifida. To investigate the potential effect of expression levels of the selected HOX genes (HOXA1, HOXA7, HOXA9, HOXA10, HOXB1, and HOXB7) in anencephaly, we evaluated 10 anencephaly cranial tissues and 10 matched normal fetus cranial samples by the NanoString technique. The results showed that the expression of HOXA7, HOXA10, and HOXB7 genes was significantly upregulated in anencephaly tissues compared with that in normal tissues (*P* < 0.05) (Fig. 6a). There was no significant   statistical difference in HOXA1, HOXA9, and HOXB1. In addition, the level of CUL4B expression was decreased in anencephaly samples (*P* < 0.05) (Fig. 6a). Western blot results showed that H2AK119ub1 levels decreased in most anencephaly samples in the comparison between the ten subjects with anencephaly and the controls (Fig. 6b). Notably, significantly negative correlations of HOXA10 expression with H2K119ub1 levels were observed among the examined subjects (rHOXA10 = − 0.8, P = 0.005) (Fig. 6C). In conclusion, these findings reveal the abnormal upregulation of HOX genes, especially HOXA10, concomitant with a decreased H2AK119ub1 level in NTD fetuses.

## Discussion

Recent studies showed that two polycomb complexes, PRC1 and PRC2, collaborate to maintain epigenetic repression of key developmental in embryonic stem cells (ESCs). Within PRC1, CUL4B act as E3 ubiquitin ligases for H2AK119ub1 that mediate transcriptional repression. In this study, we found that RA treatment led to a decrease in CUL4B and attenuation of H2AK119ub1, resulting in the upregulation of Hox gene expression. We also showed that CUL4B interacted directly with RORγ and involved in the regulation of HOX genes via adjustment of the H2AK119ub1 occupancy level at HOX genes sites. In addition, upregulation of HOX genes was observed along with aberrant levels of CUL4B-mediated H2AK119ub1 in both mouse and human anencephaly NTD cases.

The epigenetic regulation of gene expression involves several interconnecting layers, such as histone modification. CUL4B can mediate the ubiquitination of histone H2AK119 and has transcriptional inhibitory activity. Deletion of CUL4B can lead to a lack of H2AK119ub and H3K27me3, thus enhancing cell differentiation [3]. In this study, we showed that CUL4B knockdown results in alterations in HOX genes expression. Furthermore, the results revealed that binding of H2AK119ub1 was decreased in HOX genes upon RA treatment. Taken together with the previous finding that CUL4B is found in a complex with PRC, these results indicate that RA-induced differentiation results in the dissociation of a complex containing H2AK119ub1 E3 ligase activity that mediates transcriptional activation of the HOX promoters.

NTDs are a group of congenital malformations of the brain and/or spinal cord caused by failure of the morphogenesis associated with neural tube closure in early embryonic development [29–34]. Research on the factors behind the pathogenesis of NTDs has focused on environmental and genetic factors [35–42]. More than 200 candidate gene mutations potentially causative of NTDs in mice have been identified. However, very few of these candidates identified in mice have been confirmed to be involved in human NTDs [43–48]. Hox genes play an important role in the differentiation of the nervous system, especially in the determination of the anteroposterior axis. In this study, we investigated the role of the abnormal expression of HOX genes in human NTD cases and mouse embryos with RA-induced NTDs. The abnormal upregulation of HOX genes was detected in both human NTD cases and mouse embryos with RA-induced NTDs (Figs. 5f, 6a). Interestingly, aberrant HOX gene expression was detected in anencephalic but not spina bifida fetuses. The occurrence of NTD phenotype involved in region-specific mechanisms and precisely gene expression, such as Hox genes, which are activated in a temporally collinear manner to drive the progressive specification of different segments. Furthermore, to analyze the expression of HOX genes, there is a need to further expand the sample size and increase the number of studied phenotypes to provide a theoretical basis for the development of useful clinical markers.

Some studies have addressed the possible role of altered histone modification in the development of NTDs. The histone deacetylases SIRT1 and HDAC4-mediated histone deacetylation have also been implicated in NTDs [49, 50]. Moreover, loss of function of the GCN5 and P300-mediated histone acetylation also has been reported to cause development defects [51, 52]. Mice with Cul4b gene knockout died at an early stage of embryonic development, which showed the importance of Cul4b [53, 54]. Environmental factors also play a role in NTDs, such as folic acid and RA. Our findings also indicated that the downregulation of CUL4B resulted in a loss of H2AK119ub1 both in RA-induced mouse anencephaly and in human anencephaly samples. This study suggests that CUL4B might be related to the mechanism behind the development of human NTDs under RA dysmetabolism.

RA participates in the regulation of many functions in mammals, such as cell differentiation and apoptosis [40, 55–60]. The binding of RA to retinoic acid receptor results in covalent modification of the N-end and tail of nucleosome histone, resulting in the formation of an active transcription complex that regulates gene expression [61–63]. RORs participate in the regulation of various physiological processes, which include the pathway to maintain energy homeostasis and the regulation of biological clock-related components [64]. Recent studies have demonstrated that RORs function as ligand-dependent transcription factors [65]. Transcriptional regulation by RORs is mediated through interaction with corepressors and coactivators, including histone acetylases P300 and CBP [66]. In this study, we found that CUL4B interacts with and negatively regulates the transcriptional activity of RORγ, suggesting that it functions as a novel corepressor of RORγ. RORγ is required for RA-induced activation of HOX gene expression in human NT2/D1 cells. ChIP analysis showed that the level of H2AK119ub1 associated with these HOX genes regulatory sites was considerably lower in cells in which RORγ was downregulated. These observations suggest that the dissociation of RORγ to regulatory regions may correlate with a less closed chromatin structure, consistent with the functional activities of RORγ.

In summary, our study showed that epigenetic modifications of H2AK119ub1 could cause abnormal Hox gene expression after exposure to RA, which may significantly contribute to development and etiology of NTDs. The present study provided novel insight into the dysregulation of CUL4B in NTDs, which was involved in the upregulation of Hox gene transcription. Therefore, study of the pathogenesis of NTDs can provide a new approach to uncovering the complexity of NTDs and for the early prevention of NTDs.

## Conclusions

In this study, we show that the H2AK119ub1 E3 ligase CUL4B is required for the activation of retinoic acid (RA)-inducible developmentally critical homeobox (HOX) genes in NT2/D1 embryonal carcinoma cells. RA treatment led to the attenuation of H2AK119ub1 due to a decrease in CUL4B, further affecting HOX gene regulation. Furthermore, we found that CUL4B interacted directly with RORγ and knockdown of RORγ decreased the expression of HOX genes along with increased H2AK119ub1 occupancy levels, at HOX gene sites in N2/D1 cells. In addition, upregulation of HOX genes was observed along with lower levels of CUL4B-mediated H2AK119ub1 in both mouse and human anencephaly NTD cases. Notably, the expression of HOXA7, HOXA10, and HOXB7 genes was negatively correlated with CUL4B levels in human anencephaly NTD cases. Our results indicate that abnormal HOX gene expression induced by lower H2AK119ub1 levels may be a risk factor for NTDs. It also highlights the need for further analysis of genome-wide epigenetic modifications in NTDs.

## Methods

### Animals

C57BL/6 mice (44007200007011, 9–10 weeks, 18–23 g) were purchased from Beijing Vital River Laboratory Animal Technology Co., Ltd., and housed in SPF cage, approved facility on a 12-h light/dark cycle. Mature male and female C57BL/6 mice were mated overnight. The vaginal plug was detected at 8:00 am on the following morning, which designated as E0.5 if the presence of a vaginal plug. NTDs mouse embryos were induced by gavage with 28 mg/kg (body weight) of RA (Sigma, USA) on E7.5 (RA is dissolved in sesame oil). On E10.5, pregnant mice were euthanized by cervical dislocation and embryos were dissected from decidual tissue and placed in ice-cold, DEPC-treated PBS. All procedures involving animal handling were in compliance with institutional guidelines on the care of experimental animals.

### Cell culture and RA treatment

SV/129 mouse embryonic stem cells (ESCs), maintained in Dulbecco’s modified Eagle’s medium (DMEM, Gibco, USA), were supplemented with 0.1 mM β-mercaptoethanol (Invitrogen, Carlsbad, USA), nonessential amino acids (Invitrogen, Carlsbad, USA), 2 mM glutamate (Invitrogen, Carlsbad, USA), 15% fetal bovine serum (Gibco, USA), and 1000 U/ml leukemia inhibitory factor (Millipore, Billerica, USA), cultured in the culture dishes coated with 0.2% gelatin (Invitrogen, Carlsbad, USA). Cells were placed in atmosphere with 37 °C, 5% CO_2_ and passaged every 2 days. NT2 cells were developed in Dulbecco’s modified Eagle medium with 15% FBS. ESCs were treated with 1 μM RA for 24 h. Cells were incubated at 37 °C/5% CO_2_ and passaged every 2 days. ESCs were treated with 1 μM RA for 24 h.

### Co-immunoprecipitation assay

Transfection was performed using Lipofectamine 2000 (Invitrogen). Cells were harvested 24–48 h after transfection and lysed in Beyotime lysis buffer (0.5% NP-40, 50 mM Tris, pH 7.6, 120 mM NaCl, 1 mM EDTA, 1 mM Na3VO4, 50 mM NaF, and 1 mM β-mercaptoethanol) supplemented with protease inhibitor cocktail (Roche). For immunoprecipitation, 800 μg lysates were incubated with the CUL4B antibody 2 μg for 3–4 h at 4 °C followed by 1-h incubation with Protein A/G Sepharose beads (Thermo Fisher Scientific). The resulting immunoprecipitates were washed three times in HEPES lysis buffer (20 mM HEPES pH 7.2, 50 mM NaCl, 0.5% Triton X-100, 1 mM NaF, 1 mM dithiothreitol) before being resolved by SDS-PAGE and immunoblotted with indicated antibodies.

### RNA interference

- CUL4B-siRNA interference (5′–3′): GCAGCAGGUGGAUCGAAUAUTTAUAUUCGAUCCACUGCUGCTT
- RORγ-siRNA interference (5′–3′): CCCGAGAUGCUGUCAAGUUTT.

### Western blotting

The blots were incubated with the primary antibody, mouse anti-H2AK119ub monoclonal antibody (1:1000, CST, USA) and mouse anti-H3 monoclonal antibody (1:1,500,000, CST, USA) overnight at 4 °C, and then incubated with secondary anti-mouse HRP-conjugated antibody (1:5000, Santa, USA) for 1 h at room temperature. The blots were developed with SuperSignal West Pico Chemiluminescence Substrate (Thermo, USA) and quantitated on densitometer (Bio-Rad, Universal Hood II, USA) using Quantity One software.

### Chromatin immunoprecipitation (ChIP) analysis

ChIP assays were performed using the SimpleChIP enzymatic chromatin IP system (Cell Signaling, California, USA) following the manufacturer’s protocols. Chromatin was prepared, sonicated to DNA segments between 200 and 1000 bp, and then immunoprecipitated with anti-H2AK119ub (CST, USA). The immunoprecipitated DNA was analyzed by qPCR, which was performed using QuantStudio 7 Flex with SYBR Green detection. The primers used for ChIP assays are shown in Additional file 5: Table S4. Mouse IgG antibodies were used as negative controls in the immunoprecipitations. The following equation was used to calculate percent input = 2% × 2^ (CT) 2% input sample − (CT) IP sample.

### RT-qPCR

Total RNA was extracted using the Trizol method (Ambion, USA); first-strand synthesis was done with RevertAid First-Strand cDNA Synthesis Kit (Thermo, USA). Maxima SYBR Green/ROX qPCR Master Mix (Abm, Canada) was used for qPCR, and the procedure was as follows: (50 °C, 2 min) × 1 cycle; (95 °C, 10 min) × 1 cycle; (95 °C, 15 s; 60 °C, 30 s; 72 °C, 30 s) × 40 cycles; collect fluorescence at 72 °C. Primer sequences are shown in Additional file 6: Table S5.

### Luciferase reporter assay

RORE luciferase reporter assay was carried out as described previously [67]. Human NT2 cells were co-transfected as indicated with pCMV-Flag, pCMV-Flag-CUL4B, siRNA-CUL4B, and a pGL4.27-(RORE)5 reporter plasmid containing 5-RORE, using Lipofectamine 2000 (Invitrogen). After 24-h incubation, the luciferase activities were measured by a dual-luciferase assay system (Promega) and luciferase detection kit (YuanPingHao). All transfections were performed in triplicate and repeated at least twice.

### Extraction of nucleoprotein

Core histone proteins of cells were extracted using acid extraction. Briefly, cells were first homogenized in lysis buffer (10 ml solution containing 10 mM Tris–HCl with pH 8.0, 1 mM KCl, 1.5 mM MgCl_2_, and 1 mM dithiothreitol (DTT)) and chilled on ice. 5% of protease inhibitors were added immediately before lysis of cells and chilled on ice for 30 min, and nuclei were isolated by centrifugation (1500*g* for 5 min). For the preparation of histones, nuclei were incubated with four volumes of 0.2 M sulfuric acid (H_2_SO_4_) for overnight at 4 °C. The supernatant was precipitated with 33% trichloroacetic acid (final concentration) and followed by centrifugation (12,000*g* for 5 min at 4 °C). The obtained pellet was washed with cold acetone and subsequently dissolved in distilled water. Nucleprotein extraction was extracted from mouse and human brain samples using kit (Sangon Biotech) according to the manufacturer’s protocols.

### Mass spectrometry

The digested peptides were separated using a Thermo Scientific EASY-nLC 1000 System. Peptide mixtures were loaded onto a self-made C18 trap column (Acclaim Pepmap100 column, 2 cm × 100 μm, C18, 5 μm) in solution A (0.1% formic acid) and then separated with a self-made capillary column (EASY-Spray column, 12 cm × 75 μm, C18, 3 μm) with gradient solution B (100% acetonitrile and 0.1% formic acid) at a flow rate of 350 nL/min. The separated peptides were examined in an Orbitrap Fusion mass spectrometer (Thermo Scientific). The spray voltage of the ion source was set to 2.1 kV. Full-scan mass spectra were acquired in the MS over 35–1800 m/z with a resolution of 70,000. The HCD spectra resolution was 17,5000. The normalization collision energy was set to 29%.

### Immunohistochemistry

The mouse brain tissue was soaked in 4% paraformaldehyde to make the tissue fully infiltrated. Forty-eight hours after washing with PBS, ethanol is added to dehydrate, paraffin-embedded, and sliced. After washing, it was dissolved in ethanol and then placed in double evaporated water for 10 min. After rinsing the slices, the tissue antigen was repaired. We performed immunohistochemical staining for CUL4B and H2AK119ub on the same paraffin-embedded tissue blocks that were used for clinical diagnosis. Immunohistochemistry was performed using the avidin–biotin complex (ABC) method (Vector Laboratories), including heat-induced antigen-retrieval procedures. Incubation with polyclonal antibodies against CUL4B (dilution 1:100; OriGene) and H2AK119ub (dilution 1:100; CST) was performed at 4 °C for 18 h. Quality assessment was performed on each batch of slides by including a negative control in which the primary antibody was replaced by 5% BSA to preclude nonspecific signals. Pathologists who were blinded to the sample origins and the patient outcomes assessed staining. The final immunoreactivity score was determined by the Bioinformatics analysis software.

### Immunofluorescence

For the detection of subcellular localization by immunofluorescence, after fixed with 4% paraformaldehyde and permeabilized in 0.2% Triton X-100 (PBS), cells were incubated with the indicated CUL4B and H2AK119ub antibodies (dilution 1:50; CST) for 8 h at 4 °C, followed by incubation with TRITC-conjugated or FITC-conjugated secondary antibody (dilution 1:200; Zsbio Commerce Store) for 1 h at 25 °C. The nuclei were stained with DAPI (Sigma), and images were visualized with a Zeiss LSM 510 Meta inverted confocal microscope.

### Human samples

All clinical samples were from the Lvliang area of Shanxi Province in northern China with informed consent from the patients or their families. The enrolled pregnant women were diagnosed by trained local clinicians using ultrasonography and then registered (Additional file 7: Table S6). The surgical procedures were performed as previously described [68]. The epidemiological method was described in detail in our previous publication [69].

### NanoString

The NanoString nCounter was used to detect the number of transcripts in human brain tissues. Total RNA was extracted following the manufacturer’s instructions (miRNeasy Mini Kit, Qiagen), and gene-specific probes were designed by the manufacturer (NanoString Technologies). Hybridizations were carried out according to the nCounter Element 24-plex Assay Manual. Approximately 100 ng of each RNA sample was mixed with 20 μl of nCounter Reporter probes in hybridization buffer and 5 μl of nCounter Capture probes for a total reaction volume of 30 μl. The hybridizations were incubated at 65 °C for approximately 16 h, then eluted, and immobilized in the cartridge for data collection, which was performed on the nCounter Digital Analyzer. Gene expression data were filtered using quality control (QC) criteria according to the manufacturer’s recommendations. Raw counts of QC-passed samples were normalized using three reference genes as internal controls (GAPDH, CLTC, and GUSB). All QC and normalization procedures were performed using nSolver Analysis Software v2.0; all data were log2-transformed before further analysis. The Student’s *t* test was used to compare normalized expression values between normal and NTDs.

### Statistical analysis

The experimental data were analyzed by SPSS 22 statistical software. Three independent experimental data are collected. First, the normality of the experimental data is analyzed. In the case of normal distribution of the data, the statistical description is carried out by the mean ± SD, and the independent sample *t* test is used for statistical analysis. If the experimental data do not conform to normal distribution, one-way ANOVA is used. When *P *< 0.05, it was statistically significant.

## Additional files

**Additional file 1: Fig. S1.** CUL4B inhibits the expression of HOX genes in human NT2/D1 cells. **a** Quantitative RT-PCR analysis showed that CUL4B loss had a positive effect on the RA-induced increases in differentiation genes mRNA levels in NT2 cells. Data were shown as mean ± SD (*n* = 3). *P < 0.05. **b** Quantitative RT-PCR analysis showed that CUL4B loss had a negative effect on HOX genes in NT2 cells. Data were shown as mean ± SD (*n* = 3). *P < 0.05. **c**,** d** ChIP assays of H2AK119ub1 were performed using NT2 cells after knockdown of CUL4B; IgG was used as control. Enrichment of the Hox genes promoter  or unrelated region were measured by qPCR. Data were shown as mean ± SD (*n* = 3). *P < 0.05. **Figure S2.** RA-induced Hox genes expression correlates with a decreased level of promoter H2AK119ub1 in mouse ESCs. **a** Hox genes mRNA in mouse ESCs treated with RA was measured by RT-qPCR. RA 1 μM, 24 h. Actb was used as control. Data were shown as mean ± SD (*n* = 3). *P < 0.05. **b** ChIP assays of H2AK119ub1 were performed using mouse ESC after RA treatment; unrelated region was used as negative control. Enrichment of the Hox genes promoter was measured by qPCR. Data were shown as mean ± SD (*n* = 3). *P < 0.05. **c** ChIP assays of H3K27me3 were performed using mouse ESC after RA treatment; unrelated region was used as negative control. Enrichment of the Hox genes promoter was measured by qPCR. Data were shown as mean ± SD (*n* = 3). *P < 0.05. **Figure S3.** CUL4B interacts with RORγ and alterations in HOX genes expression in human NT2/D1 cells. **a** GO Analysis of CUL4B PPI interactions. **b** Expression level of CUL4B was measured by Western Blotting after knockdown of RORr **c** Quantitative RT-PCR analysis showed that RORγ loss had a negative effect on Hox genes in NT2 cells. Data were shown as mean ± SD (*n* = 3). *P < 0.05. **d** ChIP assays of H2AK119ub1 were performed using mouse ESC after CUL4B loss and RORγ; IgG was used as negative control. Enrichment of the Hox genes promoter was measured by qPCR. Data were shown as mean ± SD (*n* = 3). *P < 0.05. **Figure S4.** Western blot of luciferase reports experiment about Fig. 4a–c. **a**–**c** RORE luciferase activity was measured by Western blot matching to Fig. 4a–c.

**Additional file 2: Table S1.** CUL4B PPI_interactions new.

**Additional file 3: Table S2.** GO CUL4B PPI _interations.

**Additional file 4: Table S3.** KEGG CUL4B PPI_interation_pathway.

**Additional file 5: Table S4.** ChIP-qpcr primer.

**Additional file 6: Table S5.** RT-PCR primer.

**Additional file 7: Table S6.** Nanosamples.

## Abbreviations

ChIP: chromatin immunoprecipitation
Hox: homeobox gene
NTDs: neural tube defects
PRCs: polycomb protein complexes
RA: retinoic acid
RNA-seq: RNA sequencing
RORγ: retinoic acid-related orphan nuclear receptor gamma
NT2/D1: NT2 human embryonic carcinoma cells
