# Supplementary figures and images for: Abnormal level of CUL4B-mediated histone H2A ubiquitination causes disruptive HOX gene expression
Source: Epigenetics Chromatin. 2019 Apr 16;12:22. doi: 10.1186/s13072-019-0268-7 (PMC6466687; doi:10.1186/s13072-019-0268-7)

# Supp Figure 1

NT2

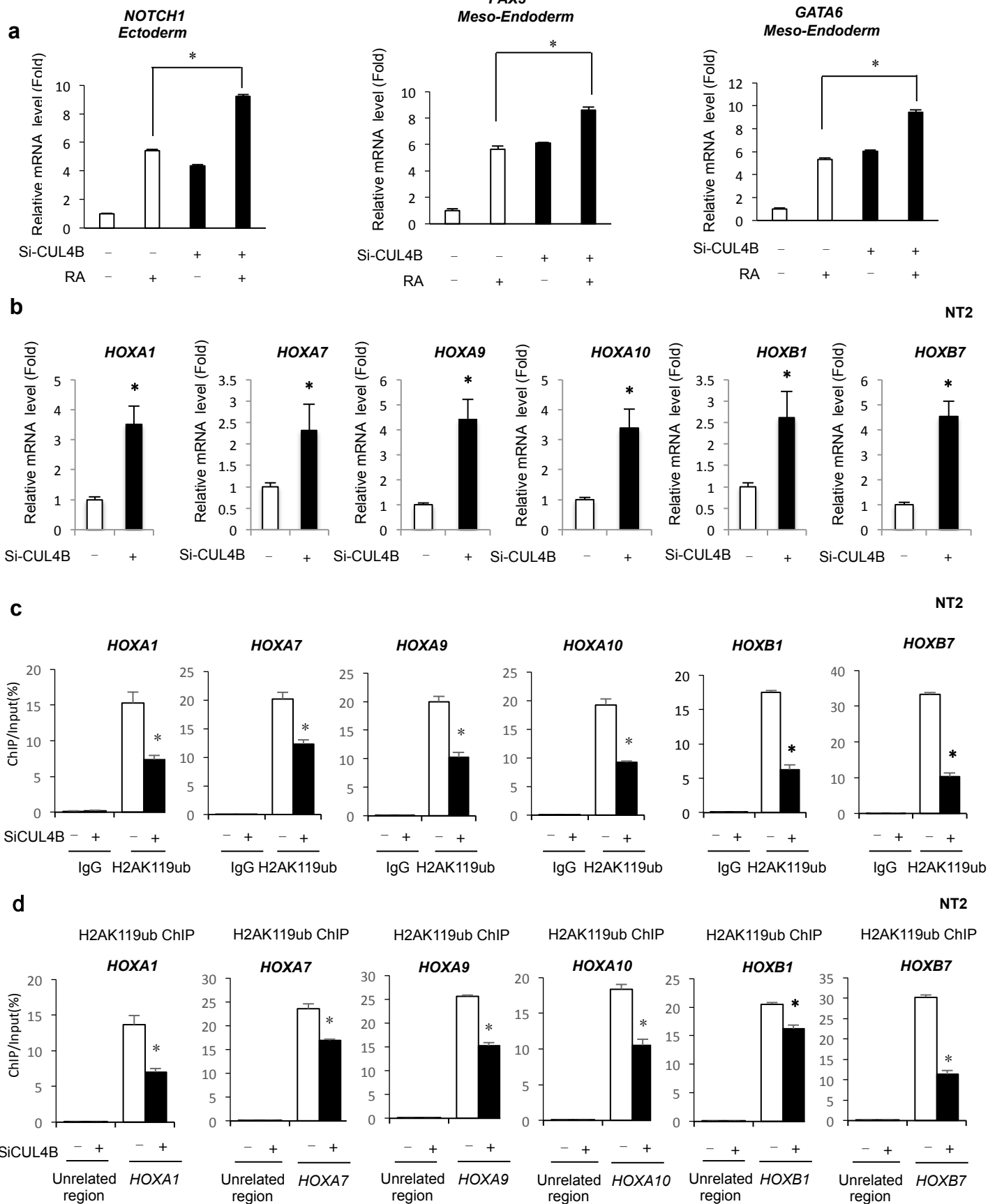

# Supp Figure 2

mESC

**a**

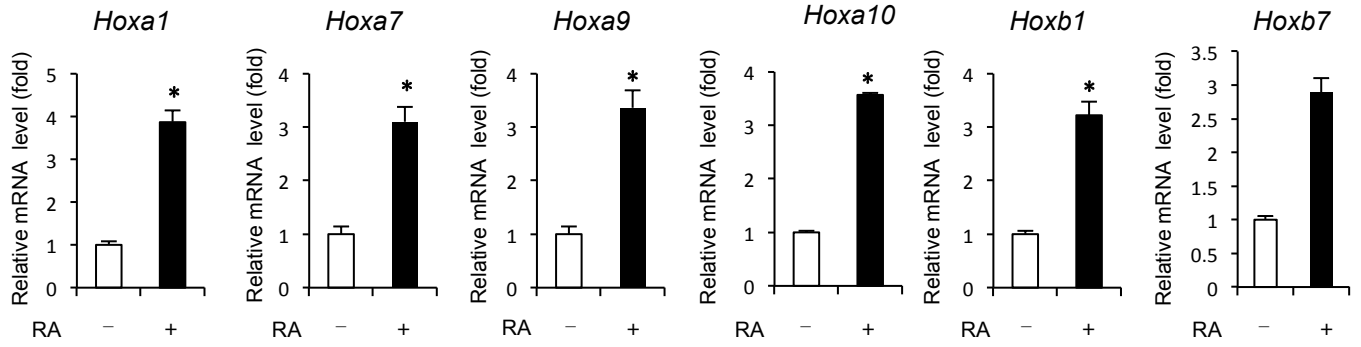

**b**

mESC

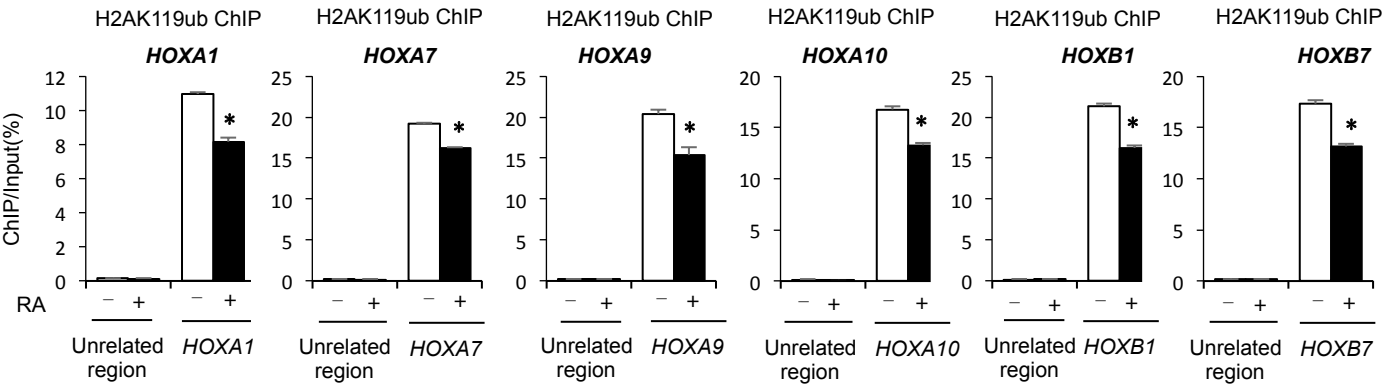

**c**

mESC

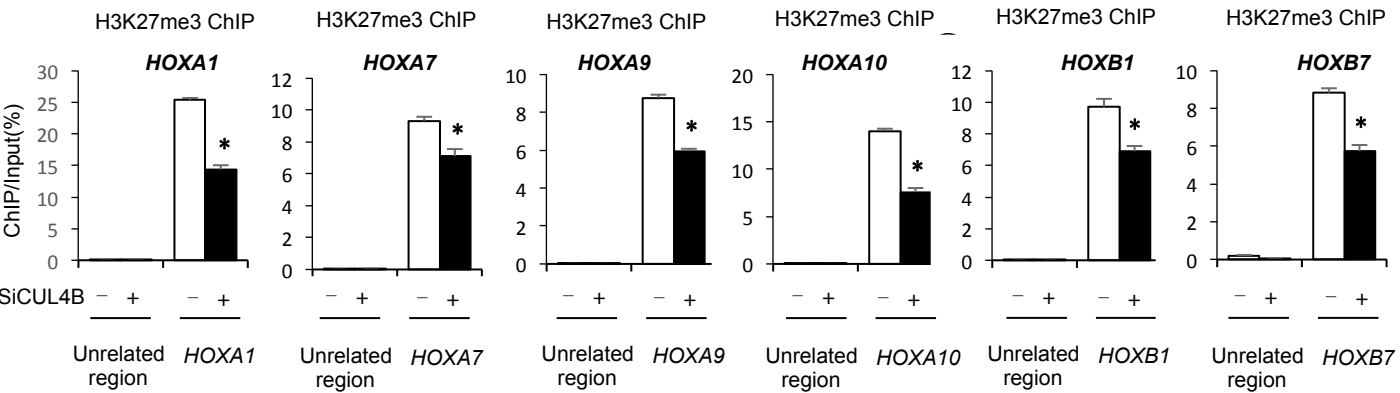

# Supp Figure 3

**a**

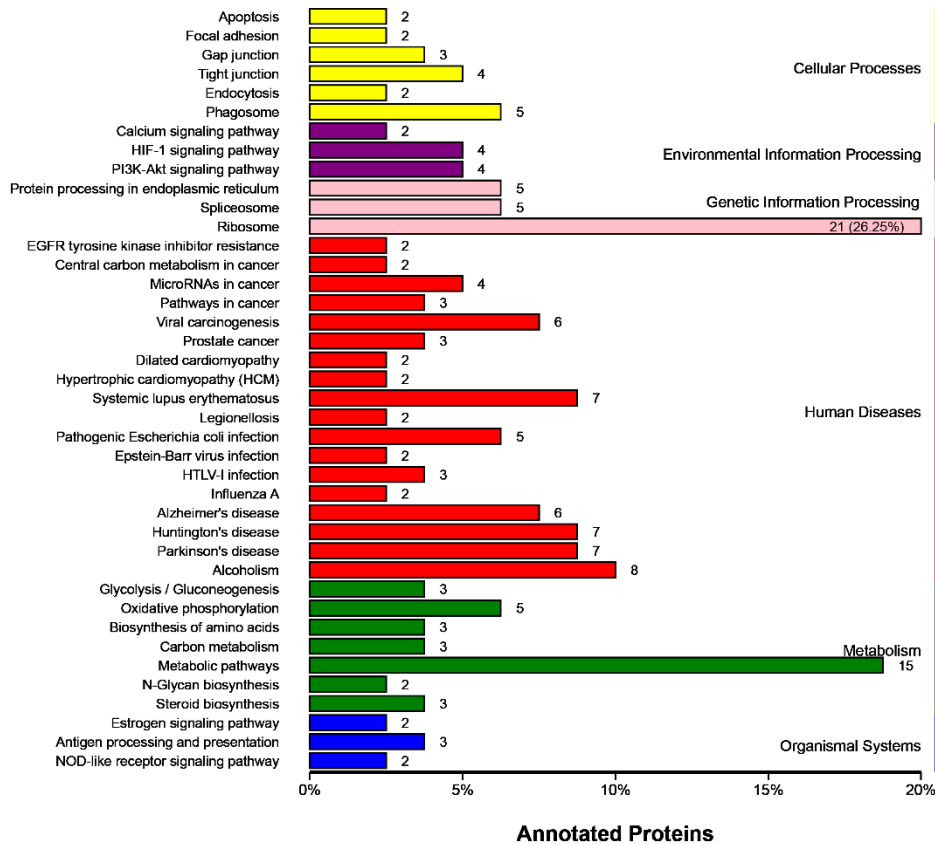

**b**

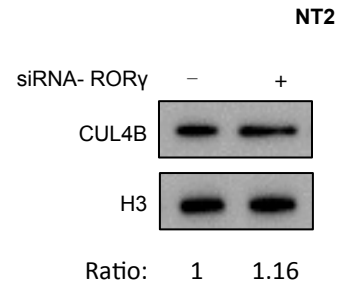

**c**

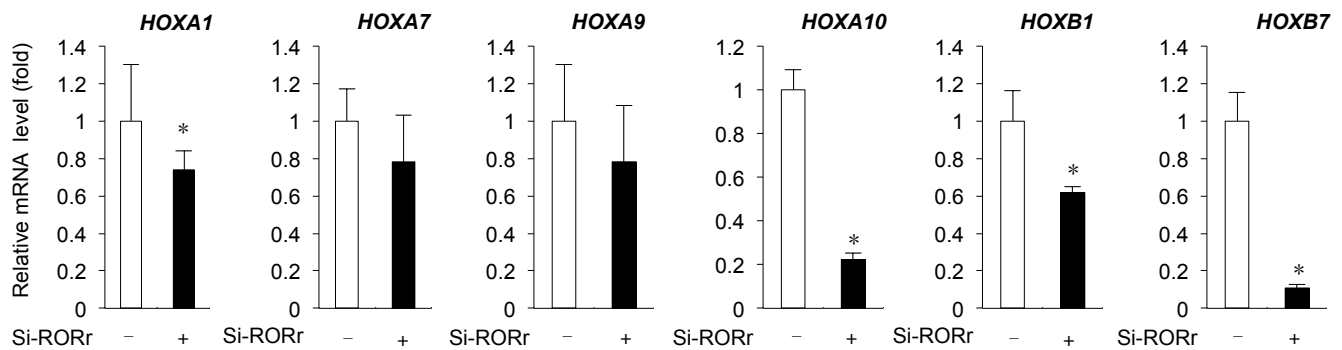

**d**

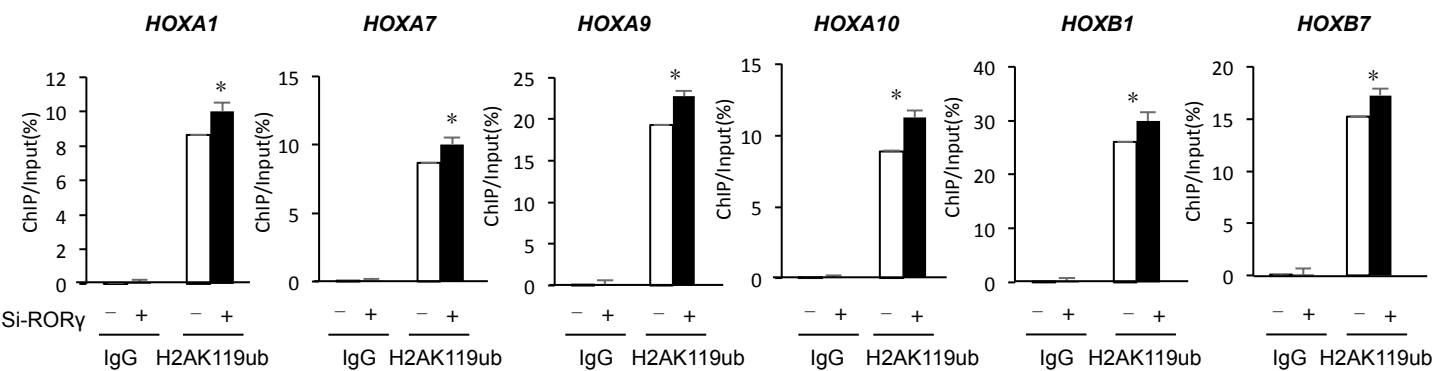

Supp Figure 4

**a**

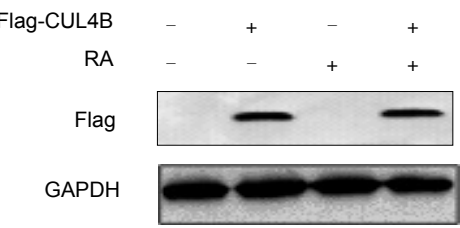

**b**

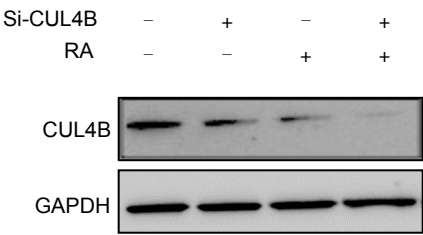

**c**

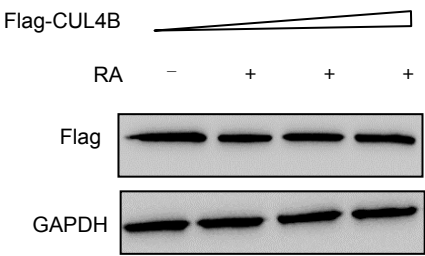

Supplement: Supplementary file 1 — Additional file 1: Fig. S1. CUL4B inhibits the expression of HOX genes in human NT2/D1 cells. a Quantitative RT-PCR analysis showed that CUL4B loss had a positive effect on the RA-induced increases in differentiation genes mRNA levels in NT2 cells. Data were shown as mean ± SD (n = 3). *P < 0.05. b Quantitative RT-PCR analysis showed that CUL4B loss had a negative effect on HOX genes in NT2 cells. Data were shown as mean ± SD (n = 3). *P < 0.05. c, d ChIP assays of H2AK119ub1 were performed using NT2 cells after knockdown of CUL4B; IgG was used as control. Enrichment of the Hox genes promoter or unrelated region were measured by qPCR. Data were shown as mean ± SD (n = 3). *P < 0.05. Figure S2. RA-induced Hox genes expression correlates with a decreased level of promoter H2AK119ub1 in mouse ESCs. a Hox genes mRNA in mouse ESCs treated with RA was measured by RT-qPCR. RA 1 μM, 24 h. Actb was used as control. Data were shown as mean ± SD (n = 3). *P < 0.05. b ChIP assays of H2AK119ub1 were performed using mouse ESC after RA treatment; unrelated region was used as negative control. Enrichment of the Hox genes promoter was measured by qPCR. Data were shown as mean ± SD (n = 3). *P < 0.05. c ChIP assays of H3K27me3 were performed using mouse ESC after RA treatment; unrelated region was used as negative control. Enrichment of the Hox genes promoter was measured by qPCR. Data were shown as mean ± SD (n = 3). *P < 0.05. Figure S3. CUL4B interacts with RORγ and alterations in HOX genes expression in human NT2/D1 cells. a GO Analysis of CUL4B PPI interactions. b Expression level of CUL4B was measured by Western Blotting after knockdown of RORr c Quantitative RT-PCR analysis showed that RORγ loss had a negative effect on Hox genes in NT2 cells. Data were shown as mean ± SD (n = 3). *P < 0.05. d ChIP assays of H2AK119ub1 were performed using mouse ESC after CUL4B loss and RORγ; IgG was used as negative control. Enrichment of the Hox genes promoter was measured by qPCR. Data were [file 13072_2019_268_MOESM1_ESM.pdf]
