# Supplementary material for: Abnormal level of CUL4B-mediated histone H2A ubiquitination causes disruptive HOX gene expression
Source: Epigenetics Chromatin. 2019 Apr 16;12:22. doi: 10.1186/s13072-019-0268-7 (PMC6466687; doi:10.1186/s13072-019-0268-7)
Supplement: Supplementary file 5 — Additional file 5: Table S4. ChIP-qpcr primer. [file 13072_2019_268_MOESM5_ESM.docx]

| Gene | Forward Sequence（5’—3’） | Reverse Sequence（5’—3’） |
| --- | --- | --- |
| *HOXA1* | TTCTCCGGCCCCATGG | GAGTGACCTGGTCCTGCGAA |
| *HOXA7* | CAGGGCTCACTAGCAGGAGTC | GGCAAGAGGCTCAAATATGC |
| *HOXA9* | TACGCGTTATTGTTCTGCTGCTGGACG | GCCCGACCCACGGAAATTATGAAA |
| *HOXA10* | GCGGGTTTGATTTCTGAGCCCTAT | GGGTTTATAGCGGCGCATTCCAAA |
| *HOXB1* | GGTCAGGCCATGGATTCGAG | GGTTACAGAGTGGGTACTCTAAG |
| *HOXB7* | TTCCACATTACCGGGAGCCGT | TTTGGCCGGATGATTTGTAGGCAG |
| *Ho*xa1 | AAGCGACAGAGAAGGAGAGA | GGCAATGGGAGCTTCTGTAA |
| *Hoxa7* | CCATTGTGAAGTCGGGTTTATGA | CAGGCCATGCTGGAAGACT |
| *Hoxa9* | GCACTGGACTTGAGCTGTAGTTTG | CCCCTGCCTTGGTTATCCTT |
| *Hoxa10* | AGAGCCTGCCCTGTTTATTT | GTGCTTCTCAGTGTGTCTGT |
| *Hoxb1* | CCGCTTAGCCCATTGGCCT | TAGGAAGGGGCTAGGGAGTG |
| *Hoxb7* | CTCGGCCTTCCCATTCATTAT | CCTCGGCCAATGGTATGAC |
