# Supplementary material for: Abnormal level of CUL4B-mediated histone H2A ubiquitination causes disruptive HOX gene expression
Source: Epigenetics Chromatin. 2019 Apr 16;12:22. doi: 10.1186/s13072-019-0268-7 (PMC6466687; doi:10.1186/s13072-019-0268-7)
Supplement: Supplementary file 6 — Additional file 6: Table S5. RT-PCR primer. [file 13072_2019_268_MOESM6_ESM.docx]

| Gene | Forward Sequence（5’—3’） | Reverse Sequence（5’—3’） |
| --- | --- | --- |
| *CUL4B* | TGCTGCTCAGGAGGTCAGATC | TGGAATCAAAGTCTTCTCTCTCGTT |
| *Cul4b* | AAACAGCAGCAGTAGCAGCA | GCAGAAGGACGAGGTTGAAG |
| *GAPDH* | ACCCAGAAGACTGTGGATGG | TTCAGCTCAGGGATGACCTT |
| *Gapdh* | AAGAAGGTGGTGAAGCAG | GAAGGTGGAAGAGTGGGAGT |
| *GATA4* | CGTTCTCAGTCAGTGCGATGT | TTGGAGCTGGTCTGTGGAGA |
| *HOXA1* | CGCACCAACTTCACTACCAA | ACTTGGGTCTCGTTGAGCTG |
| *Hoxa1* | ACCCCCAGACGGCTACTTAC | GGGAACGACAGGCTTCTTG |
| *HOXA7* | ACTTCAACCGCTACCTGACG | GTCGGACCTTCGTCCTTATG |
| *Hoxa7* | GAAGCCAGTTTCCGCATCTA | AGGTAGCGGTTGAAATGGAA |
| *HOXA9* | CCACGCTTGACACTCACACT | CGCTCTCATTCTCAGCATTG |
| *Hoxa9* | GAAGAAGCGATGCCCCTAC | TTTCGGTGAGGTTGAGCAG |
| *HOXA10* | ACACGAAGCACCAGACACTG | TCACTTGTCTGTCCGTGAGG |
| *Hoxa10* | CTGCCCTTACACGAAGCAC | TCAGTTTCATCCTGCGATTCT |
| *HOXB1* | ACCGACGAATGAAGCAGAAG | GACTGGTCTGAGGCATCTCC |
| *Hoxb1* | CATCAGCCTACGACCTCCTC | GGTGGGTTTCTCTTGACCTTC |
| *HOXB7* | GACCTACACCCGCTACCAGA | TTGATCTGTCTTTCCGTGAGG |
| *Hoxb7* | ACCGAGTTCCTTCAACATGC | AAGTCCGAGTCCCTCTGCTC |
| *NESTIN* | GGACCAAGAACTGGCTCAGG | TCTGTGGCATTCAGCTCTCC |
| *OCT4* | GGTGGAGGAAGCTGACAACA | GGTTGCCTCTCACTCGGTTC |
| *PAX6* | TCAGAGAAGACAGGCCAGCA | CATGGAGCCAGATGTGAAGG |
| *RORγ* | AGCGGCAACAGCAGCAACAG | CAGGCAGGTCAGGCGAGGAG |
