# Supplementary material for: Abnormal level of CUL4B-mediated histone H2A ubiquitination causes disruptive HOX gene expression
Source: Epigenetics Chromatin. 2019 Apr 16;12:22. doi: 10.1186/s13072-019-0268-7 (PMC6466687; doi:10.1186/s13072-019-0268-7)
Supplement: Supplementary file 7 — Additional file 7: Table S6. Nanosamples. [file 13072_2019_268_MOESM7_ESM.docx]

| Clinical phenotype | Tissue | Gender | Gestational Weeks |
| --- | --- | --- | --- |
| Normal | Brain | Male | 21 |
| Anencephaly | Brain | Male | 21 |
| Normal | Brain | Male | 18 |
| Anencephaly | Brain | Male | 18 |
| Normal | Brain | Male | 20 |
| Anencephaly | Brain | Male | 20 |
| Normal | Brain | Male | 20 |
| Anencephaly | Brain | Male | 20 |
| Normal | Brain | Female | 18 |
| Anencephaly | Brain | Female | 18 |
| Normal | Brain | Female | 24 |
| Anencephaly | Brain | Female | 24 |
| Normal | Brain | Female | 25 |
| Anencephaly | Brain | Female | 25 |
| Normal | Brain | Female | 20 |
| Anencephaly | Brain | Female | 20 |
| Normal | Brain | Female | 20 |
| Anencephaly | Brain | Female | 20 |
| Normal | Brain | Female | 18 |
| Anencephaly | Brain | Female | 18 |
